# Book Reviews

**Published:** 1816-10

**Authors:** 


### CRITICAL ANALYSIS

### *OF RECENT PUBLICATIONS*

IN THE

### DIFFERENT BRANCHES OF PHYSIC, SURGERY, AND MEDICAL PHILOSOPHY.

---

*Commentatio de Tracheitide Infantum Vulgo Croup Vocata cui Praemium a quondam Imperatore Napoleone propositum ex dimidia parte delatum est.* Auctore IOHANNE ABRAHAMO ALBERS, Medicinæ et Chirurgiæ Doctore, &c. &c. Leipsiæ, 1816.

BY the title the reader will perceive, that for the above Essay, the prize offered by the Ex-Emperor Napoleon, was divided between the author and one of his competitors. The question was proposed in the year 1807, but not decided on till 1812. The other successful candidate was the learned Jurin, Professor at Geneva. His paper, if printed, has not reached us. The one before us is dedicated to the London College of Physicians, which is the more flattering, as England seems the only country from which the author does not claim a title.

Respecting Dr. Albers's piece, we have first to remark, that, from the time that Michaelis published his inaugural dissertation on the same subject, we have not met with so copious, so learned, or so well arranged a treatise on this formidable disease. The author, like Michaelis, labours under the disadvantage of being unacquainted with the Hunterian discoveries relating to inflammation; but even their slow development, we shall find, has secured the present writer from one important error in his predecessor.

In the proœmium an apology is made for some imperfections which Dr. A. would gladly have remedied, but that the president of the commission appointed to examine the pages, expressly directed that the piece, if published, should be *dans l'etat pur virginal, si vous me permittez l'expression, ou ce memoire a ete jugê par la commission. C'est une piece justificative pour nous, &c.* Dr. Albers has, therefore, contented himself with adding copious notes, and rendering his Latin somewhat more elegant. This last alteration, we would gladly have excused. The only use of writing is to express our meaning; and, on subjects so important as health, elegance of diction is of little consequence compared with perspicuity. We have, however, met with nothing ambiguous, or at all difficult in the language beyond the proœ-

mium, mium, many parts of which are more elaborate, and consequently more obscure than is necessary or convenient.

The first enquiry demanded by the commission is, *an exact and characteristic Description of all the Stages of the Disease denominated Croup.* The answer commences with the names, learned and vernacular, given to the complaint by every author, ancient and modern, who has hinted any thing which can be construed into the disease. In this, we were surprised to find his diligence more successful than Michaelis's, who, we conceived, has exhausted the subject. Among the vernacular names, which are almost confined to the northern regions, we shall only mention those of Britain. The author supposes the disease, called in England, the *rising of the lights*, to be croup. We need make no apology for differing from him in our own language. The rising of the lights, a term we believe confined to the London bills of mortality, is, we conceive, *angina pectoris*, though, to the disgrace of our metropolis in its register, there is no precise means of ascertaining that question. The *croup* of Scotland, he derives, on the authority of Cheyne, from the word *roup*, adding, that the origin may probably be traced from the French *roupie;* but the difference between that complaint and croup is so great, that, considerable as the intercourse at one time was between those two nations, we can scarcely admit such an etymology. The author does not seem aware how little distinction there is in the full gutteral pronunciation of the northern kingdom, between croup and roup, a difference which none but a native can distinguish or imitate. Home, to whom we owe the introduction of the first word, brought it from the northern parts of Scotland. Cheyne probably followed the pronunciation of Edinburgh. This part of the chapter concludes with a rejection of all the terms, learned and vernacular. On this occasion, we think it right to give the author's own words.

"Sed credo, hoc mihi concedi oportere, ut omnes has appellationes rejiciam, videlicet minus aptas et de singulis symptomatibus desumtas, simul neque naturam morbi, neque partem imprimis ægrotam, perspicue significantes. Nihil tamen necesse est, illas denominationes singulatim examinare: igitur sufficiat, earum vanitatem uno vel altero exemplo ostendere.

"Cæteris autem usitatior est illa, quam Murraius et Michaelis in libris laudatis proponunt; anginæ polyposæ quidem seu membranaceæ. At vero habent illæ triplicis erroris materiam. Nam angina (die Bräume) recte tantummodo ac proprie dicitur de inflammatione instrumentorum deglutitionis, vix autem ad instrumenta respirationis superiora (laryngem et arteriam asperam) traduci debeat: quantum paullo ulterius demonstrabitur. Nec apte hæc angina, liceat interim hoc vocabulo uti, polyposa et membranacea nominatur: materia enim quæ in arteriam asperam effunditur, non polypum aut membranam efficit. Similiter Cynanches nomen locum invenire non potest, quod probatissimis scriptoribus inflammationem viarum deglutitionis significet.

"Neque melius suffocatio stridula morbus ille appelletur: quæ singulare duntaxat symptoma indicat, in hoc morbo nequaquam semper deprehendendum. Idem sentiendum est de voce Croup, quæ ad summum eo valet ut sonitum tussis* huic morbo propriæ, quodammodo agnoscas.

"Ex mea quidem opinione *Tracheitis infantum,* omnium aptissima denominatio est; utpote qua certe morbi natura ac sedes exprimatur, tum vero etiam ætas ei maxime obnoxia notetur."

There is something plausible in these objections, and we are ready to admit the term *tracheitis,* but the addition of *infantum* is either unnecessary or improper; unnecessary, if the disease is really confined to infants, and improper if found at any other age. If a trivial name must be assigned, we should venture to propose Tracheitis *Homeii,* from respect to the writer who first brought this form of the disease into notice. When a subject more advanced in life is seized with inflammation in this part, we would call it T. *provectiorum.* If Dr. Baillie's Cynanche Trachealis should be admitted, we should wish the two varieties, *a. Infantum vel Homeii, b. virilis, vel antiquorum.* We have already remarked how well the latter disease was described by Celsus, Aretæus, and Sydenham,† but of the former nothing can be fairly traced before Dr. Home. The remainder of this chapter is occupied in an enumeration of the changes which the parts undergo by the disease and the symptoms which attend it. The first of these was necessary to sanction the name proposed by the author; we shall, however, defer the consideration of it till we come to the chapter containing the examination of dead bodies. The symptoms are so well known, and have been so often described, that we shall pass them over, only remarking that every thing is detailed which can lead to the suspicion, or ascertaining of the disease; and, also, that the same diligence is evinced in objecting to various symptoms mentioned, but not sufficiently ascertained by certain authors. Dr. A. modestly observes, that he has never met with them, and in this we can join him without any reserve,

* We have not thought it necessary to remind our readers, but Dr. Albers should be informed, that the word Croup has its origin from the sound which issues in breathing, and not in coughing.

† See our remarks on Dr. Baillie's paper in the last volume of the Medico-Chirurgical Transactions.

particularly particularly one of the characters ascribed to the disease by Autenrieth, who boldly asserts, that no one has the disease severely more than once. On the contrary, we have observed, with many others, that this inflammation is so apt to return, and at such uncertain periods, as to render it absolutely necessary for every parent or nurse to be particularly on their guard in watching the health of a child who has once suffered the disease.

"Sed plerumque redit morbus elapso uno pluribusve imo sex annis. Qui primum morbi impetum reliquis semper fortiorem esse arbitrantur, vehementer errant, et refutantur experientia. Ipse puerum curabam, ter tracheitide correptum, cujus duo priores quidem impetus idem erant, tertius levior. Neque tamen hoc semper locum habet. Nam mecum Cheynius etiam puerum vidit, cujus morbus recidivus multo gravior erat. Commemorat quoque Borrovius infantem repetito tracheitidis accessu necatum, quod in meis ægrotis non observatum.

"Non raro accidit, ut plures infantes unius familiæ simul tracheitide afficiantur. Quod ipse in hac urbe quater observavi, tametsi semper duos tantum ejusdem familiæ curabam. Olbersius quatuor infantes ejusdem domi hoc morbo correptos vidit, quorum unus septies eum expertus, absente illo, meæ curæ postremo tradebatur. Idem testatur Cheynius. Et in America hoc frequentissime locum habere Archerus observavit. Perinde Gregorius commemorat in prælectionibus suis familiam, cujus filii omnes tracheitidem passi. Sic Des Essartesius tres infantes unius familiæ hoc morbo necari vidit. Denique Rumseius simile exemplum narrat trium fratrum, quorum duos intra tres septimanas perdidit, tertium restituit."

On the remark concerning the numbers seized in the same family, we shall have more to say when we arrive at the chapter *de causis morbi.* The remissions which some authors fancy a conversion into the intermittent form of disease are most judiciously dwelt upon. The remissions are, in fact, nothing more than what we meet with in most acute inflammations, a mere pause, because high action can no longer be kept up; they are soon succeeded by still more severe exacerbations. When the coagulated lymph is thrown out, indeed, all the symptoms will sometimes continue milder, because a new action has been set up which is one termination of inflammation. This is all very well expressed by the author, had he omitted the term *spasm.*

"Remissionibus autem non semper confidendum esse scio. Nam ab initio morbi, si accuratius attendimus, plerumque remissiones plus vel minus manifestas conspiciemus; at nullum levamen inde adducitur. Postea vero si progressus est morbus rariores et obscuriores illæ fiunt, simul autem graviores, indicando pacatam inflammationem et imminutam vim spasmorum, qui arteriam asperam tenebant et mutatam naturam materiæ effusæ vel secretæ."

The

The next question required by the Institute, is the antiquity of the disease, viz. *Whether it was described with satisfactory accuracy by the ancient writers, or by any before the last century.* Here our author shows the same learning and perspicuity as in every other part of his paper, and at the same time confirms our remark relative to the nomenclature, namely, that it is necessary to mark two species, if we add a trivial name to either.—" Exstant (says he) multæ descriptiones tracheitidis adultorum (infantes enim minus observabant), quam nonnulli cynanchen veram vel cynanchen laryngeam, Boerhavius anginam inflammatoriam et alii aliter appellabant."—What is this but admitting the necessity of a name not confined to infants? we should rather prefer *provectiorum* to *adultorum*, because most of the cases on record, particularly those contained in Dr. Baillie's communication and remarks in the last volume of the Medico-Chirurgical Society, were in men somewhat advanced beyond the meridian of life. In referring to the ancients, we find, besides those we mentioned in our review of that paper, satisfactory quotations from Hippocrates, N. Piso Fernelius Hildanus, Riverius, Ettmuller, Bonetus, A. Spigelius, and Severinus; in all of whom, the Tr. *provectiorum* may be readily recognised; but it is only in the three last named that we meet with any thing like the spurious membrane. Spigelius, as we learn from Bonetus, saw the trachea of four persons after dying under *orthopnœa*, much inflamed " ob materiam tenuem atque biliosam ipsi infarctam." That this must mean a coating of coagulated lymph can scarcely admit of any doubt. The yellow tinge which usually attends effused lymph and serum, gave the ancients the idea of bile, and the term *ipsi* [*tunicæ*] *infarctam* can admit of no other explanation. The only thing wanting in this account is the age of the patient, and this is supplied in the extract from Severinus. This author, speaking of an epidemic which suffocated young people by abscess, adds,

" ' Pervestigata larynx crustacea quadam pituita, facie exteriore contecta, citra ulceris speciem. Mortuus est *puer* octennis, septimo post die, anhelus et anxius recto plurimum capite cubans. Mihi quidem angina illa sicca visa est ab Hippocrate descripta. L. progn. com. 13. part. 16. et ab Aretæo I. acutorum." '

It should be remarked, that both these writers practised in the northern part of Europe. Hippocrates and Aretæus, to whom Severinus refers, probably never saw Tr. *infantum*. We admit that the *origin* of the symptoms (inflammation of the trachea) is the same in both ages, but the *symptoms* are different, the immediate causes of which we endeavoured to explain in our remarks above alluded to. This chapter concludes with some general observations on those authors who have hinted at the disease when treating of other complaints in the throat, and particularly the exanthemata.

An enquiry follows, *Whether Angina Membranacea was formerly as common in the Northern Regions as it is now described?* to this, Professor Albers makes no difficulty in answering, that the disease probably always existed in the same degree, though Home had the merit of first drawing public attention to its name, and the peculiarity of its symptoms.—We need not add how perfectly we agree with him. To the question, Whether the disease is more common to the north of Paris than in that metropolis, the affirmative is given with equal readiness.

A long chapter follows on the diagnostic symptoms, and particularly those by which they differ from the pulmonary catarrh, and the various species of angina. We have perused the answer to this with peculiar satisfaction; it would be less interesting to an English reader, because the pathology by which it is supported, is here pretty well understood; we are glad it is making its way to the continent.

The term pulmonary catarrh seems to have puzzled our author, at which we are not displeased; it is too much like the compound terms introduced by medical writers, when they are incapable of explaining themselves. M. Albers, however, treats it with his accustomed candour. All his other remarks show the closest observation and the greatest willingness to admit the descriptions of others where they are admissible, with a steadiness in maintaining his own which could only be excused by the fidelity of each.—*O! si sic omnia.* The close of the chapter is rendered obscure for reasons which we shall not attempt to ascertain. The disease is admitted to be well known among adults, and the case of Washington *(nunquam nimis plorandi)* is brought among others. It is said, that in some of these the new membrane was seen.—Why has not the author told us in which? and referred us to the history and description of the symptoms, with his own arguments in their support. The consequence of not pursuing this subject with sufficient minuteness leads him to the following, in our opinion, unsatisfactory conclusions.

"Ceterum variis argumentis ductus, morbum ætate provectis non tantum periculum afferre quam infantibus arbitror. Nam primum in adultis minor copia lymphæ plasticæ secernitur, unde fit ut aliæ quoque inflammationes v. c. cerebri et pulmonum eos in tantum discrimen non adducant. Quod infantibus accidisse plures cadaverum sectiones me docuerunt. Deinde illi non tanta irritabilitate tracheæ præditi, spasmodicam ejus constrictionem rarius patiuntur. Quæ Lerojus dicit: 'Le conduit de l'air est plus étroit dans dans l'enfance; sa membrane est molle, et ses nerfs, qui s'y répandent, sont plus pulpeux.'

"Denique, quod caput rei est, inde major pernicies infantibus movetur, quod in his insignis angustia sit rimæ glottidis. Hoc probarunt Lerojus, et Richerandus, et ille quidem jam aute aliquot annos in opere citato. Atque his experientissimis viris adsentitur Sœmmeringius de anatomia meritissimus, qui mihi scripsit se eandem opinionem jam abhinc multos annos habuisse. Sed quod ait Richerandus: 'Entre le larynx et la glotte d'un enfant agé de trois ou douze années, les différences de grandeur sont très-peu rémarquables, presqu' imperceptibles, et ne peuvent point se mésurer par la stature des individues:' id mihi aliter se habere videtur; refutatum quidem per explorationem externam colli.

"Ceterum censeo atque expertus sum pueros magis quam puellas tracheitidi obnoxios esse. Quod partim per diversam formam tracheæ et laryngis in utroque sexu explicandum est, jam hoc vitæ tempore conspicuam; partim derivandum a majore proclivitate puerorum ad inflammationes et causis, quæ occasionem morbo præbent apud pueros frequentioribus. Sunt etiam tracheitidi subjecti infantes robusti et sanguinis pleni, præsertim vero ii, qui per obesitatem et laxitatem fibræ sæpius catarrho aliisque morbis membranæ pituitosæ adficiuntur. Illos morbus repente aggreditur cum suffocationis periculo conjunctus. In his catarrhi signa tracheitidem præcedere solent."

It is impossible not to remark, in the first place, that the disease, so far from being less dangerous in advanced age, appears by every account to be much more so. Not to mention the formidable expressions of Hippocrates, Celsus, and Aretæus, has it not proved fatal to Washington, to two English physicians about the same age, and to most others in whom it was not instantly arrested, and even to some in the treatment of whom no time was lost? This greater danger, we conceive, may be accounted for, by the progressive increase of inflammation without any change of action. In infants, the change of action is the effusion of lymph, after which, as we observed, a remission will sometimes occur. To the term *plastic* lymph, we have no other objection than that it has a poetical air, and supersedes inquiry. The cause of less coagulated lymph being effused under inflammation of mucous membranes in advanced age, is a consideration we shall reserve for the conclusion of our remarks. The greater narrowness of the glottis, whether it exists or not, is not worth enquiry in the present instance, because no structure of this kind will have any effect in producing the adventitious membrane.

The third section is on—*the Causes which induce the Croup?* This comprehends four questions: First—*Are there well-ascertained circumstances which concur in spreading the croup more generally in one country than another?* The answer refers principally to the season of the year, and the humidity of the climate: the situation in life is considered as producing but little influence. Secondly—*With what epidemic diseases does croup commonly concur?* The answer is, that whatever state of air induces catarrh in adults, induces croup among children. Thirdly—*Is it epidemic?* This, one should conceive, answered in the reply to the former inquiry. Fourthly—*Is it contagious?* The authorities on both sides are given with much impartiality. After a host of the opposite opinion, we have six in support of the contagious property of croup: three of these are German, one English, and two American. The latter speak with much certainty; but we know how generally the transatlantic opinion of the contagious property of yellow-fever at one time prevailed. The only English writer of this opinion is the ingenious Mr. Field, the present apothecary to Christ's Hospital, who suggests something of the kind in the Memoirs of the Medical Society. But Mr. Field is by no means decided.* Fifthly—*Is croup ever the consequence of any other disease, particularly of the exanthemata?* The answer to this shows great nicety of observation in the author. He admits a difficulty of breathing, and even the effusion of lymph in a membranous form; but urges that the general symptoms, particularly the cough, the universal anxiety and the sound in respiration, are different. In small-pox, he remarks, it is uncommon, less so in measles, and most frequent of all in scarlatina. Before we dismiss this section, we shall call our reader's attention to a passage which we wish him to keep in his mind in attending to our concluding remarks.

"Quod nobis (says he) non mirum videbitur considerantibus mutationes, quæ aliorum organorum inflammationibus inferuntur per corporis ipsius rationem. Similiter peritonitis et pneumonia, quæ ubique secretioni lymphæ plasticæ favent illa puerperis, hæc morbillis juncta in infantibus præcipue secretionem commemoratam procreant. Et quem fugit diversa oculi inflammatio observata in exanthematicis morbis vel hominibus scrophulosis et post suppressam repente gonorrhœam? Quibus hominibus etsi omnibus vasa sanguinem vehentia inflammata sunt, tamen diversæ eorum in singulis affectioni peculiaria signa tribuenda, et propriam quisque curationem requirit."

We shall only notice, in this passage, the difficulty and surprise expressed by the author at the similar appearance and effect of inflammation, from whatever caused, if seated

---

* Mem. Med. Society, vol. iv. p. 151, and vol. v. p. 176. We have reason to believe that Mr. Field's ample scope of experience in Christ's Hospital has rather lessened his opinion of contagion in croup.

in the same or similar parts. Thus the inflammation from measles and from the puerperal fever are similar, if seated in similar membranes. We wish he had remarked, that, though this effusion of coagulated lymph is the common consequence of inflammation in the pleura peritonitis and other similar membranes, yet that it does not usually take place in inflammation of the mucous membrane. When these throw out coagulated lymph, it arises from the intensity of the inflammation, or from the nature of the cause. Scarlatina is always more or less attended with this kind of inflammation, if the throat suffers at all. What is called the slough, in Cynanche maligna, or ulcerous sore throat of Dr. Fothergill, is only coagulated lymph; and, when the spurious membrane is formed, it is only because the effusion has been more general and regular, instead of being, as is more commonly the case, in specks, which soon slough, and are afterwards separated by ulceration. When the specks are superficial, they slough, and ulcerate so rapidly, that the disease is called either the putrid or ulcerous sore throat. Dr. Heberden took notice of this membrane in scarlatina, which he describes as covering the uvula, and extending to the bottom of the trachea. Mr. Hunter makes the same remark. The issue of the whole is, that inflammation from other causes may, by its violence or peculiarity, produce an exudation of coagulated lymph on this as well as any other mucous membrane; yet it will not be so circumscribed in its extent, nor attended with the other symptoms of strong local irritation equal or similar to croup.

The next inquiry is into the relative mortality of croup. Here, as we may suppose, the whole, or almost the whole, is shown to depend on the period at which the disease is attacked.

"Si vero medicus in principio morbi advocetur, ægroti salus si quæ usquam datur, jure a medicis postulatur.

"Quæ cum ita se habeant, plerique ægroti moriuntur, opinor, quia advocatur medicus justo serius i. e. morbo jam consummato. Neque certa medendi ratio constat, nec detegatur unquam, qua tales ægroti utique sanari possint. Quam ob rem jure Burtonius* contendit, inter centum, quibus tracheitis consummata evenerit vix unum sanari."

The fifth section is that which we have all along kept in view. Pleased as we are with the author's diligence, candour, and good sense, we have every moment to regret how

"* Medical and Physical Journal 1800. in versione hujus libri germanica, pag. 303."

little little the Hunterian pathology is known on the continent. Most gladly would we enlarge upon it in this place, but the quantity of previous introductory matter required forbids us to do more than make use of the author's remarks in leading the reader to the grand discoveries of our illustrious countryman. This we can do with more facility in the present instance, because Dr. Albers has not encumbered us with Bichât's phraseology of serous membranes, and other equally ill-defined expressions.

The question proposed is—

"*Quelle est la nature de la concrétion muqueuse qui donne naissance à la fausse membrane qu'on observe après la mort, et qui forme les tuyaux, que l'on rend quelquefois pendant la vie?*

"Quænam est natura concretionis mucosæ, pseudo-membranam producentis quam post mortem conspicimus, et fistulas quæ interdum ab ægrotis ejiciuntur?"

"*The nature of that mucous concretion which produces the pseudo-membrane seen after death, or those cylindrical tubes thrown up during life?*" Such a question, to one unacquainted with the Hunterian discoveries, might well produce the following apostrophe:

"Ecce gravissimam mei libelli partem, quæ nostri morbi naturam multis dubiam egregie illustrat."

After viewing the opinions of others with much accuracy, the author shows that the new membrane can be nothing but coagulated lymph. This is now so generally believed in England, that it is unnecessary to produce any arguments in its confirmation. The answers concerning the fistulous, and occasionally the polypous, appearances, we reserve for another place. The chapter very properly concludes with an enquiry, *whether a similar membrane can, by any art, be produced,* or rather *whether the trachea can be artificially excited to produce such an effect; and, if it can, what phenomena are the result of such experiments?* Before we enter on our author's experiments, we shall first introduce him to Mr. Hunter's, which exactly correspond with his, though made without any view to the pathology of croup, and, consequently, on a mucous membrane unconnected with the trachea.

Inflammation, attended with the effusion of coagulated lymph, is called by Mr. Hunter the adhesive inflammation, from its property of inducing adhesions between neighbouring parts, previously only in contact, and in its more ordinary process of uniting divided parts. This is very well understood and remarked by Dr. Albers in its effect in uniting, under inflammation, the pleura with the lungs,

 peritoneum peritoneum with the intestines, and the cells of the cellular substance to each other. This is the first stage of inflammation in these parts, which, beginning with adhesion, often proceeds to the formation of matter. But in mucous membranes the provision is different.

"In internal canals,* (says Mr. Hunter,) where adhesions, in most cases, would prove hurtful; the parts run immediately into the suppurative inflammation, the adhesive inflammation in common being excluded: such parts are the internal surfaces of the eye-lids, nose, mouth, *trachea*, air-cells of the lungs, œsophagus, stomach, intestines, pelvis of the kidneys, ureters, bladder, urethra, uterus, vagina, and, indeed, all the ducts and outlets of the organs of secretion, which all these parts mentioned may be, in some degree, reckoned, and which are commonly called mucous membranes. In such parts, if the inflammation is but slight, the suppurative in common takes place, which is almost immediate, as it is not retarded by the adhesive stage, which accounts for the quickness of suppuration of these parts in many cases. I have known a violent discharge of pus come on the surface of the urethra only a few hours after contamination. These facts are shewn us every day in various inflammations of those parts, and particularly in the gonorrhœa, cold in the nose, lungs, intestines, &c. The matter from such is generally not called true matter, or purulent, but is often so, if not always, having all the characters of pus; however this will be according to circumstances. Since those surfaces are, in general, secreting surfaces, suppuration would appear to be only a change in the secretion; and I think I have visibly seen, or could visibly trace, the one change leading into the other: the different parts, therefore, of which the pus is composed, will not always be in the same proportion, so that the matter will seem to vary from true matter towards that of the common secretion of the part, and vice versa. But this does not alter the position, for it is common to matter from a sore, and even common to our ordinary secretions. If this inflammation, which produced suppuration on those surfaces, becomes more violent, we find that it moves from the suppurative to the adhesive, and throws out the coagulating lymph. I have seen this in the intestines, often on the inside of intestines that had been strangulated in a hernia. *I have been able, also, to produce it on the inside of the vagina of an ass, by injecting a strong solution of corrosive sublimate.* This is evidently the case in what is called the ulcerous sore throat; I have seen it in the trachea; I have seen it thrown up from the lungs in branches; I have seen it in the pelvis of the kidneys, ureters, bladder, and urethra."†

---

"* I make a distinction between an internal cavity and a canal: they are very different in their construction, their uses, and also their mode of action in disease are very different."

† See Treatise on the Blood, &c. p. 241.

We

We shall not think it necessary to notice any of Dr. Alber's chemical experiments, but refer with much pleasure to his attempts at stimulating the trachea, so as to produce a membrane similar to that which appears in croup. In this he never succeeded without the most violent stimuli; and even then, as he observes, the lymph was by no means so regularly cylindrical as in croup, that it adhered more firmly, and that neither the *larynx*, *bronchi*, nor lungs partook of the inflammation, which was confined to the trachea. Let us observe how exactly this agrees with an experiment of Mr. Hunter's, made on a different mucous membrane, with an entirely different view, but producing phenomena which could not be overlooked by Mr. Hunter, and of the importance of recording which he was thoroughly aware.

"The inflammation (says he) had run so high by the stimulating injections which were used for the experiments on the vagina, that the coagulating lymph had been thrown out so as almost to obliterate the vagina, uterus, &c. [of an ass] by those adhesions which are the ultimate effects of inflammation on secreting canals, while suppuration is the ultimate effect of inflammation on internal surfaces: there were no signs of inflammation on the external surface of the uterus, which is covered by the peritoneum."*

Thus, then, croup is inflammation of the mucous membrane of the trachea, excited so high as to stimulate its vessels to throw out coagulated lymph, instead of inducing only an increased and altered secretion, according to the customary action of those membranes under inflammation. When the inflammation arises spontaneously, the lymph is thrown out with more regularity, lining the sides of the membrane, and sometimes readily separated from it. When induced artificially, by the frequent application or continued contact of some highly stimulating substance, the coagulum is thrown out, with much less regularity, and adhering stronger to the inflamed parts. It appears, also, that, though the inflammation is in both cases confined, it is much less so when artificially excited.

In this place, it is necessary to make a remark which the author has, perhaps from prudence, avoided. By Michaelis, and some other writers, the disease is called *Angina polyposa seu membranacea*. Dr. A. objects to each of these words. Whilst we are ready to admit a due preference to his own term, we are not quite satisfied with the too general manner in which he rejects *polyposa* and *membranacea*, apparently for similar reasons—" Materia enim quæ in asperam arte-

* See Treatise on the Blood, &c. p. 297.

riam riam effunditur non polypum aut membranam efficit." We should rather say, that the two diseases are distinct, though confounded by these authors. When polypi, as they are called, are thrown up from the lungs, they are the consequence of slight hæmorrhage, which is instantly suppressed by the coagulation of the blood, the coagula being rendered firmer by the previous separation of the red particles, which are sometimes coughed up before the polypi, as they are called. This disease is totally different from croup, which is, certainly with much propriety, called *Tracheitis*, the whole depending on inflammation of that part. As to the term *membranacea*, we will not quarrel about it, especially as we have required two species, in one of which no membranous substance is produced.

The remainder of this meritorious piece refers chiefly to the treatment. On this we have, on several late occasions, said so much, and so often, that our readers will readily excuse us if we dismiss it with Sydenham's apothegm—Si morbi ejusdam historiam, &c. It may, therefore, be considered a compliment to Dr. Albers's diligence and perspicuity, if we pass over this part with only remarking, that nothing is omitted to render the essay complete in all its parts.

---

*Observations, with Cases, illustrative of a new, simple, and expeditious Mode of curing Rheumatism and Sprains, without in the least debilitating the System.* By WILLIAM* BALFOUR, M.D. Edinburgh. pp. 275. Black, 1816.

HAD we any ill-will towards Dr. Balfour, or any wish to pass a judgment of condemnation on the contents of his book, we should do well to employ a committee of reviewers for its examination, who should be individually and collectively interested in the sale and swallowing of drugs. The announcement of thirty-three cases of speedy cure, and that of one of the most common, and hitherto the most tedious and intractable of all complaints, without the administration of more medicine, in all of them together, than would amount to the cost of half-a-crown, has, indeed, a most portentous sound for the interests and fate of physic. It behoves physicians too, as well as pharmaceutical men, to look about them, for fees would appear to be, equally with physic, in danger. But we will not take the alarm. There

* The author of the Treatise on the Sol Lunar, which will be noticed in our next, is *Francis* Balfour, M.D.

will will still remain enough for medicine to do, even should Dr. Balfour's positions become established, that rheumatism is to be expelled the system by rubbing, bandaging, and beating. As banter and ridicule are not the weapons of our warfare, even where we disapprove and oppose, we shall at once proceed to give our readers a candid statement of the principles and practice of the book submitted to our notice.

We must be permitted, however, in the first place, to say that Dr. Balfour, in contending against those theories of rheumatism which make it consist in lentor of the fluids, a peculiar acrimony, or a particular state of the muscular fibre, displays his ingenuity to very little purpose. The notions of lentor and acrimony are now generally exploded, nor is the opinion quite new, that the seat of rheumatism is membranous rather than muscular, and that the muscles are merely affected in consequence of their connection with their respective fasciæ and investing membranes. We think, indeed, that Dr. B.'s reasonings prove rather too much, and go, in some measure, to establish the doctrine that every other local disorder must necessarily, as well as rheumatism, have its seat in the cellular substance, since, wherever there is organization, there must, he infers, be cellular structure; and where disease exists, the organization must be affected. But, having just protested against prejudging, we hasten to present our readers with an abstract of our author's physiology, and of his pathological opinions concerning the complaint in question.

"The knowledge we possess of the functions of the cellular membrane goes a great way (says Dr. B.) in explanation of the phenomena of rheumatism. We know that it officiates at once as a fascia, a ligament, a mucous gland; and that by it is secreted all the fat and oily substance that is deposited about the joints, upon, between, and in the interstices of the muscles."

The circumstances connected with muscular contraction afford evidence that "the fasciæ of the muscles confine them to their situation parallel with the bones;" and that, thus united with the fasciæ, and the fasciæ with them, affections of the membrane by which these fasciæ are constituted must, of necessity, interfere with regular and healthy contraction. "Hence the pain and difficulty of motion, in the first stages of the disease at least, of a limb affected with rheumatism."

In the second place, the "debility, contraction, and rigidity of chronic rheumatisms," are explicable only upon the principle of aponeurotic affection, since the cellular membrane, by disease, becoming unequal to a due supply of of that lubricating mucus which is constantly required for muscular motion, an incapacity of free action, by which the chronic state of the complaint is characterized, ensues in course and consequence. The wasting, too, of a limb affected with chronic rheumatism, is to be explained in the same manner, since there comes to be a less deposit of that fatty matter by which are constituted the roundness and plumpness of muscular substance.

"In the third place, the effusions that sometimes take place into the sheaths of the tendons, is a proof that the cellular membrane is principally affected in rheumatism."

"In the fourth place, from the fact that the same cause affects different parts, of similar structure, in different individuals, and in the same individual, perhaps, at different times, we might judge, *à priori* almost, that it is not the muscular fibre, but the cellular membrane, that is peculiarly affected in rheumatism. Thus, exposure to cold produces in one person catarrh, in another pneumonia, in another pleuritis, in another rheumatism, according to idiosyncrasy. Now, the parts affected in catarrh, pneumonia, pleuritis, however they may differ in density, are of the same nature with the tendinous aponeurosis of the joints and muscles, and the cellular substance interposed between their fibres. It is, therefore, not illogical to conclude, that it is the cellular membrane, not the muscular fibre, that is primarily and peculiarly affected in rheumatism."

In the fifth place, Dr. Balfour argues, that the circumstances of sprains and spasms producing sometimes rheumatism, strengthens the notion of this latter being a membranous disorder, since the membranes are the parts that suffer in the above accidents or maladies; and sixthly, the fact of the acute species, at least, of rheumatism, attacking, in the first instance, the joints, makes it, we are told, more than probable that the capillaries of the aponeuroses and cellular membrane constitute the seat of the complaint.

Lastly comes the practical inference in support of the theory, which we shall give at length, in the words of the author.

"The mode of operation of the various means employed in the cure of rheumatism, puts it quite beyond a doubt, that the proximate cause of the disease consists in a languid or obstructed circulation in the parts affected. Heat, which is generally employed either as a principal or auxiliary remedy in the management of this disorder, whether applied externally or internally, moist or dry, whether produced by action, or accumulated in the body by clothing, cannot but stimulate the whole system. If the temperature of the atmosphere that surrounds us is increased, the body becomes larger, because the circulating fluids expand and dilate the vessels. The motion of the heart is also increased, both in frequency frequency and force, so that the whole circulating mass moves with greater velocity. If a patient, labouring under rheumatism, feels relieved by such increase of temperature, or, which is the same thing, by the accumulation of heat in his body, upon what principle does the relief thus obtained depend? Can a more natural inference emanate from any premises, than that the stimulus of heat, by rousing to action the vessels which were in a state of atony, enables them to propel their contents? We see this verified every day, upon a small scale, by the most ignorant people, who apply a hot iron to a part affected with rheumatism, till the pain is removed. Now, can it be conceived that the pain is removed in any other way than by discussing the congestion that had taken place in the capillary vessels? These observations apply to the operation of heat, from whatever source derived,—whether applied to the body externally, as by the increased temperature of chambers, or the warm bath; whether produced by motion, or accumulated by clothing; whether excited by drinking warm fluids, or diffusible stimuli.

"The medicines exhibited in rheumatism with the view of producing evacuations, cannot affect the parts on which chiefly they are intended to act, without previously, simultaneously, or by consequence, affecting the whole system. Diaphoretics cannot increase insensible perspiration, nor sudorifics produce sweat, without affecting the parts intermediate between the stomach and skin. And, neither can emetics and cathartics operate on the stomach and bowels without promoting the action of the absorbents. Tonics even, a most useful class of medicine in the cure of rheumatism and its consequences, if they do not quicken, must increase the force, at least, of the circulation.

"Now, if the remedies employed in the cure of rheumatism operate by increasing the actions of the system, we cannot avoid concluding, that the parts affected are relieved by having their action promoted: for what takes place in the system must take place in every part of it. Consequently, atony, or diminished action, must be the proximate cause of the disease. But no other vessels than the capillary can be obstructed, without being attended with consequences very different from whatever take place in rheumatism. Therefore, the mode of operation of the different remedies employed in the cure of rheumatism, proves, that the proximate cause of that disease is an affection of the capillary vessels, or of the white parts.

"These are the data (says Dr. Balfour, in conclusion,) on which I have endeavoured to found an entirely new mode of treating rheumatism, and other complaints allied to it, as illustrated in the following cases."

Before presenting our readers with one or two of the most remarkable of these cases, it may not be improper to make the same observations in respect to our author's claims to practical novelty that we have before advanced in reference to his theorizing. That Dr. Kellie's proposal of pressure from the tourniquet, as a remedy for rheumatism, was an anticipation of Dr. Balfour's system of bandaging, we will not pretend to assert, because the principle of treatment, in either case, is not altogether similar; but surely friction, and other mechanical stimuli, had been thought of and practised long before our author favoured the world with his present treatise: hence we do not think he is justified by facts in denominating his plan "an *entirely* new mode of treating"* the complaint. Whether it be new or old, however, is of subordinate consequence to whether it is likely to be attended with general success in the hands of individuals who have no system to support, or no pre-conceived notions to ratify.

"I was led (says Dr. B.) into the above speculations by what occurred in my own person, and which I have since practised upon others with almost uniformly the same good effect. Having been seized with a rheumatic affection of the left shoulder, chiefly in the course of the deltoid muscle, the pain, at times, but especially towards morning, when warm in bed, was so severe as to make me cry out. Desirous, on one of these occasions, of moving my arm, a task to which its own powers were unequal, I grasped it firmly with my right hand, about the middle of the pained muscle. To my surprise and high gratification, I was instantly relieved from pain; and while I thus held my arm, could do any thing with it I pleased, without further aid from my right hand than mere compression. This, therefore, was the remedy, the only remedy to which, on all future occasions of the kind, I had recourse, and it never was employed without success.

"I now began to think that surely the muscular fibre was not the seat of pain in rheumatism, not even of those pains occasioned by motion. If it were, how could mere compression enable it to contract with all its pristine vigour? I observed, moreover, that when, during the paroxysm of pain, I endeavoured to move my arm, the moment the belly of the muscle began to press on the aponeurosis, I was obliged to stop; but, as soon as artificial resistance was opposed to it, the muscle could perform its functions with the utmost ease. A more decisive proof, I think, cannot be adduced, that the pain and difficulty of motion of a limb afflicted with chronic rheumatism are not referable to the muscular fibre. It may occur to the reader, as it did to me, that the sudden relief from pain which I experienced is to be explained on the principles of ligatures interrupting the progress of pain, or any other sensation, along the course of the nerves, as in some cases of epilepsy, whitlow, &c. But this by no means accounts for the fact; for I always remained free from pain a considerable time after compression was removed." Case 1st, p. 28.

Dr. B. will not, it is to be hoped, conceive our criticisms to to be in the determined spirit of cavil, when we further venture to hint, that the effects of pressure continuing after the cause had ceased to be applied, is by no means demonstrative that such pressure operated without any reference to nervous influence: as well might he contend that the progress of the epileptic aura is not interrupted upon this principle, because the menaced paroxysm does not take place even after the removal of the preventive means. Indeed it may be made a question whether a good deal of what is remedial in rheumatism may not become so, as least as far as the diminution of pain is concerned, by some primary operation upon the nervous system; and that, without at all invalidating the supposition of the membranous nature and seat of the complaint. We apprehend that, had our author, while lying on his bed under the pains of rheumatism, been subjected to some violent impulsion of the sentient organization; had he, for instance, learnt that the apartment beneath him was in flames, his affected deltoid would instantaneously have acquired as free play as any that pressure could have given to it.

But, whatever might be the principle of its operation, Dr. Balfour was very fairly led to the inference, that, as temporary pressure proved so serviceable in his own case, such pressure might be contrived of a more permanent nature, as a more permanently operative remedy; accordingly, in the second case, we find him employing bandages to the affected limbs, which were attended with the like good effect; and we do not know that we can select a better case than this second one for laying before our readers.

"On the 21st of October 1814, I was called to a girl of fourteen years of age, whom I found sitting in a warm room, before a large fire. Her skin was hot, face flushed, and pulse at ninety; all which I was disposed to attribute to the warm regimen she had been so carefully observing. I desired her to remove to a distance from the fire. She told me she could not move, on account of rheumatic pains, reaching from her ancles to the middle of her thighs. This account I soon found to be correct, for when, in consequence of my pressing her, she made an attempt to rise, she was forced to cry out most bitterly, so that I saw her limbs were totally immoveable. I told her I would make her walk through the room without pain before I left her. So much difficulty, however, was experienced in removing her from the place where she sat, that I began to suspect I had put my credit to too great a hazard, and therefore endeavoured to pass off as a joke the assurance I had given of instant relief. Having accomplished the removal of the patient to a proper situation, *I applied a roller of flannel, with a degree of tightness which she could easily bear, to*

 *both*

*both limbs, beginning at the middle of the thighs, and continuing it downwards towards the feet.* I now requested she would make an attempt to walk. To this she consented, on condition of being indulged with another person's arm. I allowed her to touch gently her aunt's shoulder. The girl rose up, and walked through the room, stifly to be sure, but without complaint. I ordered her to be put to bed, and, as she seemed to labour somewhat in her breathing, was desirous of taking a little blood from the arm. This was peremptorily resisted by her aunt, the lady of the house, on the score of the patient's youth. It was in vain I represented, that, though bandaging was a powerful auxiliary, it never could supersede the diligent use of other remedies generally employed in the cure of rheumatism; above all, that it could have no influence whatever on febrile symptoms. Next day the patient's general health was much the same; the pains as bad as ever. I suspected the cause, and soon found the bandages were hanging loose. I immediately replaced them with the same good effect as before. With the exception of some laxatives, this girl got no other remedy, and the pains were, notwithstanding, put to flight in a few days. A sister of this girl, aged eighteen, soon after my patient got better, was seized in like manner, all over the inferior extremities, with rheumatic pains. Without calling any medical aid, she had recourse to the bandages, which had the same happy effect with her as with her sister." Case 2d, page 31.

These cases are followed by several others, all more or less illustrative of the beneficial effects of bandaging; but we observe, as the author proceeds in his narrations, he some how or other comes to supersede bandages by the manual applications of friction, and more especially of percussion. Indeed the cases towards the conclusion of the treatise contain scarcely any allusion to the former, but are full of the latter method of treatment. Our limits will only allow the insertion of one of these cases, and the last of them we select almost at random.

"On the 19th of December, 1815, Mr. W. S., aged about forty, of a very full habit of body, was laid up with rheumatism, which had hung about him the preceding fortnight, and manifested itself by pains flying through his body, but affecting his knees principally. I was called to him on the 20th, and found him complaining of a violent pain in his back, striking through to his very heart, as he expressed it, upon attempting the least motion. The seat of the pain was on each side of the spine, between the under ends of the shoulder-blades, darting upwards along the right side of the neck, round the head, and down the right arm. The muscles of the neck were swelled and inflamed, and, at their insertion particularly, extremely painful to the touch. The head was, on this account, incapable of motion. Confined, indeed, by the pain in his back, to one posture, the patient was compelled to lie lie in bed like a log of wood. Pulse 80, tongue white, with a very bad taste in the mouth. In these circumstances, I was much inclined, I confess, to employ the lancet; and, had I taken 30 or 32 ounces of blood from the arm, and repeated the operation to the same amount within twenty-four hours, I would only have imitated what is at present called by some 'efficient practice.'

"My patient, however, was strongly averse to being blooded on the present occasion. I therefore had recourse to the same means, friction and percussion, which I would have employed in a rheumatic affection of the joints and tendons, and with the most immediate beneficial effect. When I entered his room, the patient could not raise his right arm from the bed; but in five minutes he put his hand to his neck, and to the crown of his head. The neck, which he could scarcely suffer to be touched, I handled gently at first, and gradually with more freedom, till the pain was sufficiently subdued to allow perfect motion to the head. This effect was produced within ten minutes of my entering the room. With difficulty I now got my patient to sit up, when I began percussion on the back, which was attended with such darting pains to the heart, and such a cutting of the breath, as the patient expressed himself, as required the utmost caution in the application of the remedy. I laid him down a few minutes, and when he was raised up again, he bore the operation much better. I laid him down a second time, and, when raised up, he bore the percussion better still. I therefore continued it some minutes, and until he hastily cried out he was relieved. I asked him in what respect he was relieved? He said the pains, both external and internal, were nearly gone; and he could breathe much more freely. These are facts—astonishing facts—which incontestibly prove that percussion can obviate incipient inflammation. I now laid my patient down, when he declared he could turn himself which ever way he pleased. These things took place at two o'clock P. M. and were all transacted in the course of half an hour. Ten drachms of sulphate of magnesia were ordered to be taken immediately. At seven o'clock was still nearly free from pain. What little remained, was seated lower down the back. Applied percussion, which occasioned very trifling uneasiness. Pulse 80. Medicine had begun to operate.

"Next day, (December 21) at eleven a. m.; pulse still 80; is free from complaint, with the exception of a slight pain at the connections of the sacrum, and which immediately gave way to percussion. Ordered an ounce of sulphate of magnesia in the evening. Seven o'clock p. m.; has been up all day, felt chilly for a short time after rising; pulse 100, which the patient attributes to hanging over the fire; no return of pain. December 22, past a restless night; head-ache in the recumbent posture, no pain otherwise; pulse 72. Ordered six leeches to the temples. Seven o'clock p. m.; head-ache gone, free from complaint. December 23; passed a good night, perfectly well.

"Thus, in less than forty-eight hours, nay, I may say, almost immediately, diately, was a rheumatic affection completely removed, which, by the ordinary mode of treatment, might have been protracted for a fortnight or a month; and the patient inevitably reduced to a state of extreme debility. The reader, whoever he is, if ever attacked with rheumatism will not long hesitate, I presume, betwixt the excessive evacuant plan of cure, and submitting a few minutes to percussion, applied under the control entirely of his own feelings."—Case 33, page 237.

The attention which we have paid to Dr. Balfour and his book, shows that we are far from suspecting any thing of charlatanism. We may, however, remark, that such is still the unfortunate uncertainty of medicine, that we find every novelty supported by a host of undeniable facts. The reader will not fail now and then to be reminded of Perkins's tractors, and animal magnetism, while perusing the statements of Dr. Balfour.

We believe, however, the principle to be in the main correct, that a proper stimulation is the thing of most promise in conquering rheumatic affections; we have ourselves witnessed abundant benefit in these complaints, not only from friction, but we think more especially from a due management of the stimulus of galvanism, which would appear to influence the system somewhat in the way of Dr. Balfour's percussion, and is most undoubtedly a far preferable stimulus to common electricity, as being at once equally if not more diffusible, and certainly more permanent. There are features so common to paralysis, and some kinds at least of rheumatic affection, as in our minds to justify Professor Duncan's appellation of paralytic rheumatism, applied to one form of the malady; and, it is in this species of rheumatic complaint especially, that we have tried and found so much benefit from galvanism; but Dr. B.'s practice would seem to extend the stimulant mode of treatment to all kinds of rheumatism, even the inflammatory and acute, as especially evidenced in his last case, and we must allow that there are certain circumstances connected with the curative plans tried in rheumatism, which would seem to favour his notions. Thus, Peruvian bark, and the ammoniated tincture of guaiacum, in large quantities, have been administered with good effect, according to the testimony of some respectable authorities, in cases where the general diathesis, as marked by the pulse and other symptoms had been high and inflammatory. In these cases, however, it must strike every one as proper to pay attention to the symptomatic irritation in the use of stimuli.

We have given this long account of Dr. Balfour's opinions and mode of treatment in a very frequent complaint, on account count of that very frequency. That rheumatism appears under a variety of forms, arises from a variety of causes, and is often cured by apparently contradictory remedies, is sufficient to show the necessity of varying our remedies, and even multiplying their number. This, by the motto we have prefixed to all our late volumes, we profess to be our principal object, this we wish always to keep in view. The most successful mode of treating the commonest diseases will gradually prevail so as to become universal; it is, therefore, particularly necessary to bring before our readers a treatment of apparently the same diseases, when they have not yielded to the customary remedies. In this view we may remark, that the perusal of the work before us may prove useful to all such as know how to add the *granum salis* to the reports of authors who are advocating a favourite hypothesis. Dr. Balfour's style is for the most part plain and forcible; now and then we find him rather too wordy, and his treatise is disfigured by the occasional occurrence of awkward expressions and many Scotticisms. The latter would not have been noticed, had we not observed in most of his countrymen, an attention very flattering to us, of confining themselves to the southern dialect.

---

*Synopsis Nosologiæ, or Outline of a new System of Nosology.* By T. Parkinson, M.D. Small 8vo. pp. 25.

Of all the reviewers in the three kingdoms, nothing could be more unfortunate than that Dr. Parkinson's work should fall into our hands. The book is elegantly printed on very fine paper; every passage speaks the scholar, the man of industry, and of genius. But from us, who, for some time, have been quarrelling with nosology, particularly with the word typhus, what mercy can Dr. Parkinson expect.

It may seem unfair to introduce the reader into the middle of a book. As, however, the author has chosen to place a preface there, as we found the previous matter extremely difficult to be understood, and as we despair of making a work so compressed more intelligible, by any abbreviation of our own, we shall content ourselves with giving Dr. P.'s address introducing his Nosology, and the preface to his Syllab[illegible]s.

"To the most learned, and the most worthy, the Professors and Admirers of Medical Science, these Tables of *Nosological Nomenclature* are *dedicated*, with the greatest deference and esteem, by their obedient servant, Thomas Parkinson, M.D. designed to facilitate and improve the study of medical science, by elevating it to a progressive, methodical, and logical system, fixed upon solid and and just principles, governed by acknowledged and inflexible laws, and divested of fanciful and delusive hypothesis.

*Leicester; June* 28*th*, 1815.

"*Address.*—These Tables will serve as part of a complete System of Zoo-nosology, which is in great forwardness, comprehended in three Tables of Anatomy, Physiology, Nosology, and Therapeutics, with proper explanations; to which will be added, Syntaxis Zoonosologica, proper to be taught methodically in the schools of medicine.—Taught at Leicester by the author."

The following is the preface to the Syllabus:

"If nosology be exalted to a system of science, it must be capable of, and demand, an appropriate nomenclature.

"An appropriate nomenclature will, in the illustration of it, develope the principles on which the system rests; to form and establish a nosological nomenclature, on a solid basis, is, therefore, to display the foundation of a system.

"It is only by assigning certain limits to the capabilities of abstract parts, and giving to each its relative value in the composition of the whole, that we can avoid confusion and error.

"Symptoms are characteristics to which they must be limited; otherwise they will invade another province, in the government of which they cannot be suffered to participate; this would be illogical; if, therefore, symptoms, which must be limited in their power to the discrimination of diseases, be allowed any share in the government of denominations, the nomenclature will be incorrect and erroneous; consequently the system will be feebly supported.

"While the necessity of a new system of nosology has been generally acknowledged, many difficulties were foreseen in the way of founding and establishing one upon solid and just principles, so that it has been almost generally believed and averred, that, to accomplish it is not within the capability of any one man: individuals, have, therefore, naturally shrunk from the task; and the different views in which men behold medical science at large, and nosology in particular, oppose seemingly insuperable obstacles to the attainment of a concordant nomenclature, by the conjoined labours of many.

"I, though an humble and almost obscure individual, have taken a different direction in the pursuit of medical science, to any of my predecessors; but, I conceive, not less luminous or extensive in its view, in which I progressively became convinced that nosology did admit of being placed upon a solid foundation; that the superstructure, both in its materials and arrangement, might be improved, and the whole so reduced to a system as to be brought under the government of laws equally inflexible and just.

"This appeared an arduous and difficult task; yet I did not despair of success in the undertaking, and began by determining the the different departments, and by what peculiarities each should be governed.

"I deemed it expedient to adhere to the departments of Cullen, viz. classes, orders, genera, species, and varieties; but have found it essential to the system to add one more department, namely, types.

"Having reduced the whole into a synoptical form, I compressed that even into tables; those tables I have explained by other tables; to these I have added a scheme of nosological syntax, and have prefixed, by way of introduction, axioms upon the laws which form and govern life, health, disease, and death, giving, I trust, an intelligible and luminous view of a system, which I am endeavouring to develope and illustrate, by publishing the whole in a *Syllabus* of a Course of Lectures on Nosology."

The Syllabus itself contains a mere enumeration of names, arranged according to order, genera, and species; after which follows Syllabus II. which commences thus:

"*Recommend the adoption of this Nosological Language in Thinking, Speaking, and Writing, on Diseases.*

"The path in pursuit of medical science will be illumined.
"The judgment will be corrected.
"Confidence will be strengthened.
"Arguments will be logical and uncontrovertible.
"Conversation will be dignified and admired.
"The profession will be exalted and reverenced.

"*Hope to obliterate the term Fever from Nosological Language.*

"Fever is never an idiopathic disease.

"Is always symptomatic of local Hyperbiŏsis;* therefore, it cannot be allowed any separate or distinct appellation.

"Typhus fever is a symptom only, or rather is composed of an assemblage of symptoms inseparable from a given type of Hyperbiosis;† the identity of the disease is inflammation of some solid of the first Order, Platymorphia, and the increased Sensibility, the increased Mobility, the increased Circulation, and the increased Caloric, the component parts of inflammation, are the evidences of its existence.

"Intermittent and remittent fevers are of the same Class as typhus fever, and of the same identical Genus, differing only in degree of intensity of local inflammation, that is, in type.

"A given intensity of Hyperbiŏsis Diffūsa Encephalīca will intermit, and the fever will correspond in intermission.

---

* Hyperbiŏsis, we learn, in different parts of the book, is the same as Cullen's Pyrexiæ, and derived from ὑπερ and βιωσις.—Edit.

† How can this agree with Cullen's typhus, particularly the latter part?—"pulsus parvus debilis; vires plurimum imminutæ."—Edit.

"With greater intensity it will remit, and the fever will correspond in remission.

"With greater intensity it will be progressive, and the fever will correspond in progression; and this is typhus fever.

"By elevating Hyperbiŏsis intermittens it becomes Hyperbiŏsis remittens: the symptomatic fever will, therefore, be remittent; and, by elevating it to progressīva, the symptomatic fever will be continued.

"I have to add, that, if what is usually called Typhus Fever terminates in death, on examination of the dead body, the seat of local Hyperbiŏsis may always be discovered, and will be found either in a highly inflamed state, or, perhaps, more commonly in a state of gangrene."

It may seem premature to criticise a syllabus, which can only profess to offer a very imperfect sketch of the author's arguments in favour of his doctrines. But, for our mutual instruction, or, if not so, for our own, we cannot help remarking, that where local action is the primary cause of fever, we usually call it symptomatic fever, and the cause is, for the most part, apparent enough. Where local mischief occurs at an uncertain period of fever, we consider it the effect of that fever, and here such effect is often very obscure.

There is only one other remark we shall make on a subject so new to us as the contents of Dr. Parkinson's book, namely, that very long observation has confirmed us in our opinion, that too close an attention to nosology has often paralyzed the efforts, or given an undue confidence, to the young practitioner; and that, as far as we have proceeded in this reformed nosology, we have reason to fear that its effect on Dr. P.'s scholars may be similar.

---

### *Edinburgh Medical and Surgical Journal*, No. XLVII. for July, 1816.

(Concluded from p. 253.)

Art. VIII.—*On Yellow-Fever.* By J. B. Sheppard, R. N.

This paper should have been superscribed, *Can a person be attacked a second time with yellow-fever; and is the yellow-fever, the Bulam, and Gibraltar or Cadiz fever, the same?* The author confines his inquiries to these questions, and conducts them with much judgment. He first shows, by a practical knowledge, acquired by eight years' service in the West Indies, that the having passed through yellow-fever is only a comparative security, depending, probably, on the being afterwards exposed to the miasma in a more concentrated form. In this manner he accounts for the exemption of the the French refugees from St. Domingo to Philadelphia, and of the European troops transferred from the West-India islands to the southern part of Europe. We are glad of this opportunity of illustrating our proposed distinction between infection and contagion. As we remarked, in answer to Dr. Dickson, the quantity of *contagious* matter makes no difference in the effect, when applied to a subject susceptible of its impression. But the concentration or quantity of miasma produces a variety in its effects which is easily distinguished by the season of the year, as well as the degree of approximation to the source. In the same manner, in the camp fever, the quantity or concentration of the causes modifies the effect, and even the susceptibility. As a proof of this, it is a well-established fact, that nurses, and medical attendants in general, who are proof against the ordinary causes of camp or hospital fever, which affect strangers, will become susceptible of the effects of air, impregnated, beyond a certain degree, with these morbific effluvia.

The question concerning the identity of Bulam, yellow, and Gibraltar fever, is considered by the author as hardly to admit of a doubt, nor has he the least scruple in determining that none of them is contagious, but all of domestic origin.

Art. IX.—*Case of violent Contraction of the Uterus, threatening Abortion, cured by copious Bleeding, and enormous Doses of Opium.* By Edward Lloyd Knowles, Surgeon, Soham, Cambridgeshire.

Of the necessity and propriety of the first of these remedies, few practitioners, we trust, at this time, need be informed. The quantity of opium may, however, be worth recording:—

| " *On the 5th June.* | *Tinctur.* | *Gum.* |
|---|---|---|
| From 8 P. M. to 12 | gtt. 160 | grs. — |
| 12 to 4 A. M. | 320 | 12 |
| 4 A. M. to 7 | 180 | 6 |
| 7 to 11 P. M. | 180 | 9 |
| 11 P. M. to 7 A. M. of the 6th | 180 | 12 |
| On the 7th | 85 | — |
| On the 8th | 60 | 39 grs. of solid opium. |
| | 1165 drops of the tincture. | |

"The first day she became indisposed, 840 drops of the tincture, and 27 grains of the solid gum, were administered."

Art.

Art. X.—*Two Cases of Wounded Artery in Venæsection, cured by Compression.* By C. K. Crawford, Surgeon, R. N.

These cases do great credit to the candour and good intentions of the writer. To the former, as the accidents happened under his own practice; to the latter, as they must prove the means of instructing his young successors to preserve a proper equanimity, under similar events. Both these cases were relieved immediately after the operation; and the artery not only healed, but remained pervious, without any communication with a vein. Our Journal affords an instance in which a complete aneurism from a wound had existed for several months, yet the artery was afterwards obliterated by pressure above the incision.*

Art. XI.—*Appearances, on Dissection, in a Case of Humoral Asthma.* By the same.

Humoral asthma is a very general term, and, like Angina Pectoris, we conceive may arise from a variety of causes. The account of the diseased part being short, we shall transcribe it, as a record concerning a complaint extremely common in our humid and cold regions.

"The cartilages of the four lower true ribs were ossified, and those of the upper of a very firm structure. The whole parietes of the thorax seemed very rigid, strong, and not well adapted for free motion. The heart was large, and of a pale colour. The right lung, except a small portion of the superior part of the upper lobe, adhered to the pleura costalis, and, from its texture, seemed to be entirely useless. There was neither air in its cells, nor blood in its vessels. It was of a pale grey colour, and collapsed into a small space.

"The cavity of the right pleura seemed diminished by the great convexity of the diaphragm towards the thorax, and the mediastinum being pushed from its natural position more towards that side. The remainder of the cavity that was not occupied by the lung, was filled with cellular substance.

"The whole of the left lung, and small portion of the right, mentioned above, were of a dark liver colour, without adhesion. On cutting into their substance, a great quantity of frothy phlegm, intermixed with a dark-coloured blood, issued out. Their whole substance seemed to be as completely filled with this fluid as a sponge well soaked in water; and it ran out in a stream, without pressure, on merely making an incision with the knife.

"The pleura costalis, except at the place of adhesion, was natural, nor was there any water in the cavities of the thorax.

"The trachea appeared very vascular, and, on its anterior side,

* See vol. vi. p. 543, where the plan is proposed which succeeded in Mr. Crawford's cases.

within the thorax, there were, betwixt its cartilaginous rings, portions of imperfect rings of extravasated blood in the state of coagulation.'

Such are the contents of the first part of this Number, which, if not equal to the last, has still a large portion of useful communication. It is not our custom to notice any thing more; but our attention to language, and the important controversy in which both the Journals have lately partaken, will not permit us to pass over the two reports at the conclusion.

"The term *Cachexia syphiloidea* (says the first reporter) has been appropriated by Mr. Pearson to a class of symptoms with which he is undoubtedly better acquainted than any other practitioner, but which have not yet, perhaps, been sufficiently investigated to be finally subjected to generalization. It expresses the leading characteristics of this polymorphous disease with brevity, and is less equivocal than *pseudo-syphilis*, and some other appellations that have been proposed, as it implies merely a malus habitus, resembling, in its external signs, the morbid state which the poison of syphilis induces. The symptoms of syphilis were soon detailed with great minuteness and accuracy, and mercury early ascertained to be its specific remedy; but it seems to have first occurred to the sagacity of John Hunter, that such symptoms were not invariably the result of the syphilitic virus, nor invariably required the specific treatment. Observation has subsequently confirmed that opinion, and greatly extended it; insomuch that the soundest observers begin to admit, that there are, in fact, no symptoms which unequivocally characterise a disease of syphilitic origin, or unequivocally indicate the necessity of administering mercury; and that it is only by combining an examination of the appearances, with a history of their origin and progress, and of the treatment to which they have already been subjected, that a satisfactory conclusion can be deduced. Neither the nodes of the periosteum, the ulcerations of the fauces, nor any of the modifications of cutaneous eruption, upon which so much stress has been laid, can be relied upon as unequivocal indications of a syphilitic or non-syphilitic disease. An ingenious attempt has lately been made, with great confidence, in the sister island, to settle the characteristic eruptions connected with particular modifications of the cachexia, resulting from specific primary ulcerations, as well syphilitic as non-syphilitic; but, anxious as every zealous practitioner must be for solid instruction upon this subject, and ready as every friend of independent inquiry to admire the acuteness of the suggestion, it is but too manifest that this attempt is premature. If the point, indeed, could be thus expeditiously settled, it would have been determined long ago. The practical inference, from this state of uncertainty in the diagnosis, however, is, that we should be extremely cautious in pronouncing diseases to be syphilitic, litic, without a careful examination of their history and treatment, and of subjecting a cachectic patient to the severity of a mercurial course, when he may be restored to health by more safe and certain means."

No one can have a higher respect for Mr. Pearson, nor are we disposed to object to the term *Cachexia syphiloidea;* but we cannot immediately perceive the advantage science can derive by its substitution for *pseudo-syphilis*, and, after regretting the *Pandora's box* of one author, the *proteiform* disease of another, and the *multiform* of a third, what must we feel at meeting with a paraphrase of the latter term in *polymorphous*, which, like the rest, we fear, will only add to the resources of indolence, in substituting words for industry, in accurately discriminating the true character of a disease now better established than of any other morbid poison.

But what we are most alarmed at is, that, after so much has been done by Mr. Hunter and his followers, an attempt should be threatened—*in chaos antiquum confundere*. It is admitted that by "combining an examination of the appearances with the history of their origin, progress, and treatment, a satisfactory conclusion may be deduced." This is strictly conformable to Mr. Hunter's direction, and to those who have followed it. Why, then, are we afterwards told, alluding to Mr. Carmichael's labours, that his ingenious attempt, as it is called, "to ascertain certain characteristics," must be premature? Has he not fixed the exact character of certain eruptions, which, till Mr. Hunter's time, were confounded with venereal? has he not ascertained their frequency, and mode of treatment? and is not this a considerable step towards the desirable object in view? In a word, is it not a step beyond the undefined and undefinable terms cachexia syphiloidea, pseudo-syphilis, pandora's box, proteiform, multiform, or polymorphous.—On this we shall probably have more to say, when we offer our remarks on Mr. Geoghegan's "Commentaries," which shall appear in our next.

In another report, we earnestly intreat the ingenious writer to tell us what is meant by *febris continua*, whether it is the same as *typhus*, or the contagious fever. We meet with the first expression in the table, and the two latter in the report; and here all the fevers, or most of them, might be called *febres catarrhales*. To make these registers useful, they should be perspicuous. We recommend Dr. Willan's Reports, which appeared first in the Monthly Magazine, and have been since published in a separate volume, as the most unexceptionable model for every future reporter.

